# Electrophysiological function of the retina and optic nerve in patients with atrial fibrillation

**DOI:** 10.1007/s10633-015-9498-6

**Published:** 2015-04-25

**Authors:** Michal Post, Wojciech Goslawski, Monika Modrzejewska, Maciej Wielusinski, Jaroslaw Kazmierczak, Wojciech Lubinski

**Affiliations:** Department of Ophthalmology, Pomeranian Medical University, Szczecin, Poland; Department of Cardiology, Pomeranian Medical University, Szczecin, Poland

**Keywords:** Atrial fibrillation, Ablation, Retina, Optic nerve, Electroretinogram, Visual evoked potentials

## Abstract

**Purpose:**

To evaluate the effects of atrial fibrillation (AF) and ablation procedures on electrophysiological function in the retina and optic nerve.

**Methods:**

Thirty two eyes of 17 patients with AF were analyzed. The full-field electroretinogram (ERG), pattern electroretinogram (PERG) and pattern visual evoked potential (PVEP) were performed. The results were compared to age-matched healthy controls (*n* = 30). In 12 eyes, electrophysiological tests were performed before and 3 months after ablation treatment.

**Results:**

Statistically significant differences between AF patients and healthy controls were detected. In the full-field ERG, a reduction in the oscillatory potentials wave index (OPs WI; *p* = 0.012) and scotopic (0 dB) a-wave amplitude (*p* = 0.009) was observed. The amplitude of b-waves, scotopic (24 dB; *p* = 0.011), photopic single flash (*p* = 0.008) and photopic flicker (*p* = 0.009), was decreased. The photopic flicker b-wave peak time was increased (*p* = 0.005). Other parameters of ERG/PERG/PVEP did not differ significantly from controls. After the ablation procedure, the only statistically significant change was an increase in the OPs WI (*p* = 0.002).

**Conclusions:**

In the analyzed series of AF patients, retinal dysfunction was detected in the ERG test. The AF ablation may improve the retinal function as indicated by an increase in the OPs WI. The OPs WI has a potential value in the estimation of the effectiveness of AF ablation.

## Introduction

Atrial fibrillation (AF) is the most common cardiac arrhythmia affecting 0.4–1 % of the population [1]. Approximately 2.2 million individuals in the USA and 4.5 million in the European Union have AF [1], and the number of patients is likely to increase during the next 50 years, in consequence of the growing proportion of elderly individuals [2, 3]. Rate or rhythm controls are the two possible methods to manage AF. The ablation procedure is nowadays a widely acceptable method of rhythm control. The essence of the AF ablation is to make an electrical isolation between the pulmonary veins (which are the most common place of AF triggers) and the cardiac left atrium [4].

In AF patients, cardiac output decreases by 20–30 % [4], causing reduction in the organs’ blood supply and generating the ischemia of the central nervous system (CNS). Therefore, the optimal effect of AF treatment should be taken into account of not only heart function, but also in case of the CNS. There are many appliances to assess improved blood flow after AF ablation at different levels: carotid ultrasonography [5], fluorescent microspheres in retinal circulation [6] and cerebral computed/radionuclide angiography [7]. However, electrophysiological tests directly measure the function of the retina and optic nerve. The inner retina has a similar oxygen consumption rate as the brain (2–5 ml O_2_/100 g min) [8] and similar oxygen tension in dark adaptation as the myocardium [9, 10]. However, the outer retina is more sensitive to hypoxemia. This is due to the fact that photoreceptors’ (especially rods’) high oxygen consumption varies between 3.9 and 5.1 ml O_2_/100 g min in dark adaptation [11–13] and is reduced by 30–70 % in light adaptation [11, 12, 14, 15]. Photoreceptors are supplied mostly from choroidal circulation, which has no autoregulation [6, 16, 17]. Consequently, the outer retina is susceptible to ischemia due to systemic hypoxia. Therefore, the outer retinal dysfunction occurs, when blood oxygen tension has value of <70–80 mmHg, while in the inner retina (supplied by the central retinal artery) the first signs begin below *p*O_2_ < 40 mmHg [18, 19]. All of these data insinuate that in patients with AF dysfunction of rods (in scotopic conditions) will occur in the first place.

The present study is the first to evaluate the influence of the cardiac arrhythmia (AF) and its treatment (ablation) on the function of the retina/optic nerve/CNS.

## Methods

Thirty-two eyes of 17 patients with AF were analyzed. They were randomly selected from the group of patients who underwent the AF ablation in the Department of Cardiology PMU. The ocular inclusion criteria were the best-corrected distance visual acuity (VA) > 0.5 (Snellen chart) and normal results of routine ophthalmological examination. Patients with diseases (e.g. diabetes mellitus, heart failure), with hemodynamically significant internal carotid artery stenosis (>70 %) or taking drugs (e.g. propranolol) with known influence on the function of the retina and optic nerve were excluded. Patients with treated hypertension (RR < 140/90) were enrolled.

The electrophysiological tests mentioned below were performed in all patients: full-field electroretinogram (ERG), pattern electroretinogram (PERG) and pattern visual evoked potentials (PVEP). All results were compared with age-matched healthy controls (*n* = 30), and parameters of the tests were as follows.

### Full-field electroretinogram (ERG)

Recordings of the flash ERG, scotopic oscillatory potentials were performed using the UTAS-E 2000 system (LKC Technologies, Inc.). The ERG test parameters were performed in accordance with ISCEV standard [20, 21] with some reduction in flash strength. The patients’ pupils were dilated with 10 % neosynephrine and 1 % tropicamide eye drops. The ERG responses were then recorded from the anesthetized cornea (0.5 % proxymetacaine) using a Burian–Allen bipolar contact lens electrode; a gold ground electrode was placed on the ear. Interelectrode impedance was maintained as <5 kΩ. A 30-min dark adaptation (20 min according to the ISCEV) preceded the rod and the mixed cone-rod recordings; a 10-min period of light adaptation preceded the photopic cone and flicker recordings. The rod ERG recordings were obtained with single white flashes (Grass Flash xenon lamp, luminance 1.58 cd s/m^2^, ISCEV: 0.025 scotopic cd s/m^2^, equivalent to 0.01 photopic cd s/m^2^) attenuated by a 24-dB neutral filter. The mixed rod-cone ERGs (maximal response) were elicited with flashes of white light (luminance: 1.58 cd s/m^2^, ISCEV: 3 cd s/m^2^). The cone ERGs were elicited with white flashes of the same intensity. The luminance of the background was 30 cd/m^2^. In the flicker recordings, the frequency of stimulation was 30 Hz (the same conditions of adaptation) and ten sweeps were averaged. The bandpass filter resulted within 0.3–500 Hz in all recordings. The oscillatory potentials in the scotopic state were obtained with white flashes (luminance: 1.58 cd s/m^2^, ISCEV: 3 cd s/m^2^). The high-pass filter was 75 Hz, and the low-pass filter was 500 Hz. Overall wave index of OPs amplitudes (O1 + O2 + O3 + O4) was measured. The artifact reject threshold was off in all flash ERG recordings, and the notch filters were off.

### Pattern electroretinogram (PERG)

The PERG was recorded with the RetiPort System (Roland Consult Instr.) using a protocol implemented in the original software of the system. Monocular stimulation was used, together with appropriate refractive error correction in relation to the eye-screen distance. Examination was interrupted when frequent blinking or fixation losses were observed (patient was monitored with a TV camera). The patients’ pupils were not dilated, and central fixation was used. Parameters of the PERG stimulation were as follows: 21″ CRT monitor with a frame rate equal to 75 fps was used; black and white reversing checkerboard (30° FOV) was presented to the patient, with a check size equal to 1°2′; temporal frequency was equal to 4.6 rps (2.3 Hz), Michelson contrast: 97 %, luminance for white elements: 120 cd/m^2^. Electrodes: ground (gold disk) electrode was placed on the forehead (Fpz), thread DTL electrode was used as active, gold disk was placed at the outer, and canthus ipsilateral was used as reference. Parameters of the recording system were as follows: amplifiers sensitivity −20 µV/div, filters: 1–100 Hz, artifact reject threshold –95 % (for the amplifiers range ±100 µV), notch filters were off, averaging: 200 sweeps, sweep time: 250 ms (time base: 25 ms/div). Two consecutive waveforms were recorded; then, they were off-line averaged and analyzed. The PERG test parameters were in accordance with the ISCEV standard [22].

### Pattern visual evoked potentials (PVEP)

Pupils were undilated. Stimulation was monocular. Eyes were refracted for the stimulus viewing distance (one meter). Parameters of the stimulation were as follows: 21″ CRT monitor with a frame rate: 75 fps was used, black/white reversing checkerboard was presented to the patient with a check size: 1°4′ and 0°16′, Michelson contrast: 97 %, temporal frequency: 2 rev/s (1 Hz), luminance for the white elements: 120 cd/m^2^ (mean luminance: 55 cd/m^2^). Electrodes: active-gold disk electrode was placed on the scalp over the visual cortex at Oz with reference-gold disk electrode placed at Fz; ground (gold disk) electrode was placed on the forehead at fpz. The electrodes were placed relatively to bone landmarks according to the international 10/20 system. Central fixation was carried out. Parameters of the recording system were as follows: filters: 1–100 Hz, notch filters were switched off, artifact reject threshold: 50 µV, sweep time: 300 ms, averaging: 100 sweeps. Two consecutive waveforms were recorded; then, they were averaged off-line and analyzed. The amplitude and time of the P100-wave with manual correction to the automatic cursor placement were also parsed. In order to evaluate the bioelectrical function of macular versus more peripheral regions of the retina stimulation with the small (0°16′) and large (1°4′), check stimuli were measured. The PVEP test parameters were in accordance with the ISCEV standard [23].

#### Cardiac ablation

The goal of AF ablation is to make an electrical isolation between the pulmonary veins-PV (which are the most common site of AF triggers) and the left cardiac atrium. The device used in this procedure was a Medtronic ArcticFront Adv. cryoballoon introduced through a femoral vein inside the left cardiac atrium by a transseptal approach. After inflating the cryoballoon, the device was placed consecutively in the ostium of each pulmonary vein. To reach low temperatures, a firm adhesion of the balloon to the PV is obligatory, which what is confirmed by injecting the contrast inside the balloon closed PV. No contrast leakage to the atrium confirms the proper placement of balloon (observed in fluoroscopy); the balloon is filled by a Medtronic Cryoconsole device with nitrous oxide which is providing low temperatures (often below –50 °C) on the balloon surface. The electrical conduction block was checked with a ten-pole lasso electrode placed inside each vein. During the whole procedure, each patient was heparinized by monitoring of the anticoagulation time (ACT) and adjusting the heparin dose targeting by 250–350 s.

In 12 eyes of the patients, additional ERG/PERG/PVEP examinations were performed after successful ablation. The mean follow-up period was 89 days (62–115 days), and during the follow-up there were no significant modifications of drugs among patients. Decrease in the group size (*n* = 32 to *n* = 12) resulted from the ineffectiveness of the ablation procedure, poor general condition of patients or lack of cooperation during the test.

#### Statistics

The Shapiro–Wilk test was used to evaluate the compatibility with normal distribution of analyzed electrophysiological parameters. The ERG/PERG/PVEP parameters in eyes of patients with AF and eyes of the control group were compared in the following way: (1) For normally distributed data, the arithmetic mean and standard deviation were applied, and Student’s *t* test was used (2) for non-normally distributed data, the Wilcoxon signed-rank test was used. The results were considered as normal when they included between mean and ±2SD (*x* ± 2SD) (normally distributed data) or between 2.5 and 97.5 percentiles (non-normally distributed data). Two parameters (amplitude and implicit time of waves) in following groups were analyzed:

1. AF patients before ablation versus control group,
2. AF patients before ablation versus AF patients after ablation.

Four hypotheses were tested; there was post hoc adjustment of *p* values for multiple testing (Bonferroni correction). The significance level was *p* < 0.0125 (0.05/4), and for statistical calculations, the number of eyes tested was used, rather than the number of subjects.

## Results

**Fig. 1:**
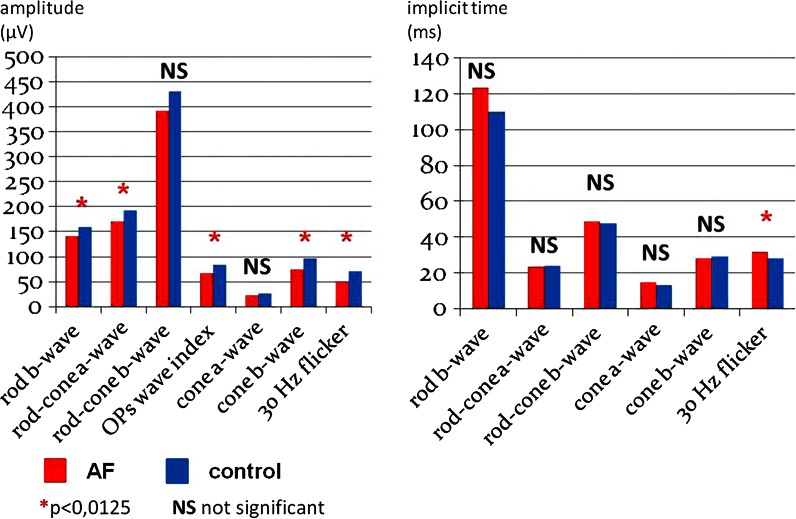
Full-field electroretinogram—eyes of patients with AF (*n* = 32) in comparison with controls (*n* = 30)

**Fig. 2:**
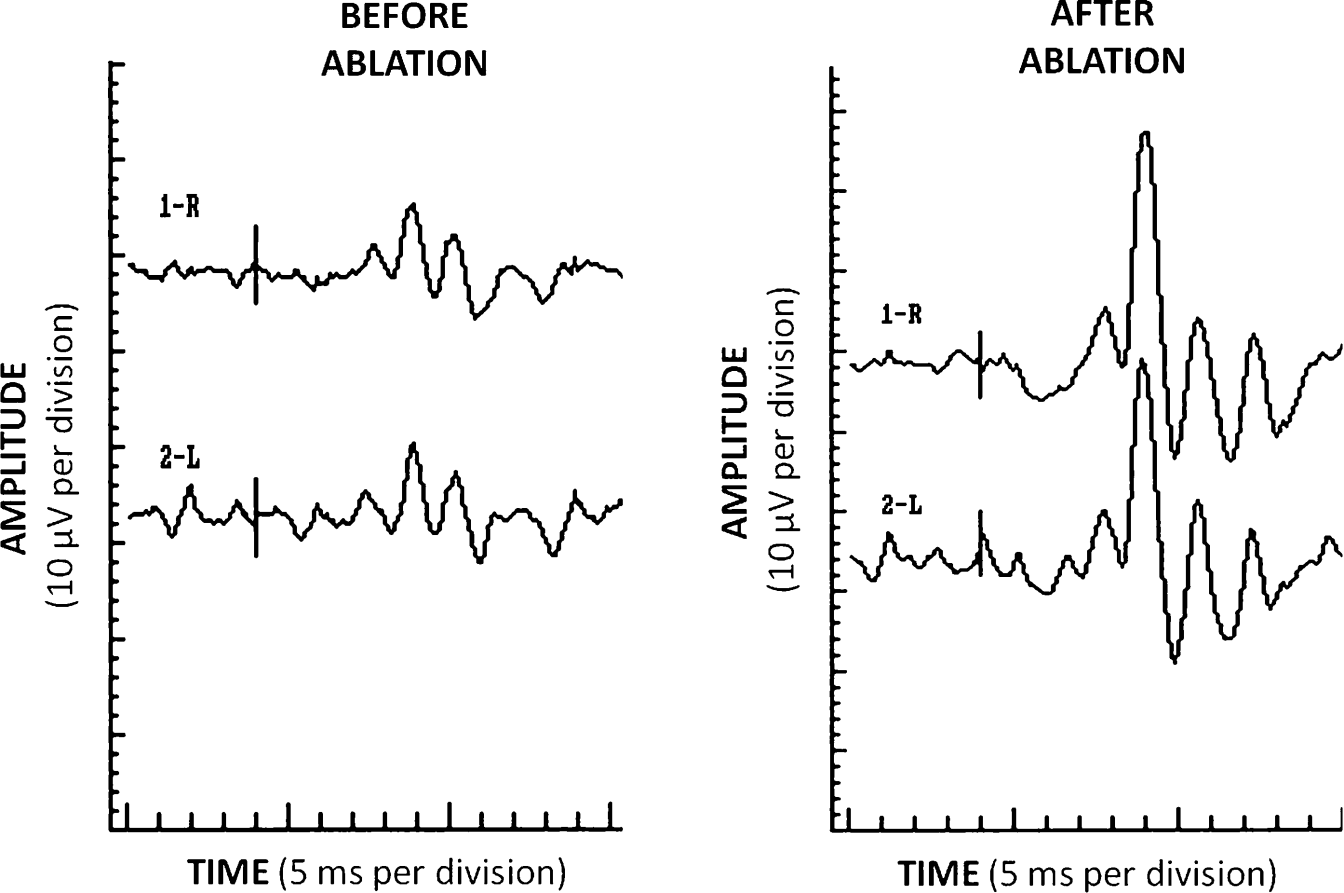
Reduced OPs WI of eyes of patient with AF and its improvement after ablation

Results of the statistical analyses (ERG) of AF patients in comparison with controls are shown in Table 1 and Figs. 1 and 2. Thirty-two eyes of 17 patients with AF (12 women and 20 men) were analyzed; the mean age was 63.2 years ± 7.8 (range of age 53–83 years). Eleven patients (*n* = 22 eyes) had treated arterial hypertension, and eight (*n* = 16 eyes) were smokers. No other systemic diseases were observed. The best-corrected visual acuity was 0.71 ± 2.2 (Snellen chart).

**Table 1:** Full-field electroretinogram—eyes of patients with AF (*n* = 32) in comparison with controls (*n* = 30)

	Amplitude (µV)					Peak time (ms)				
	AF	*N*	Controls	*N*	*p* value	AF	*N*	Controls	*N*	*p* value
Rod b-wave	140.9 ± 79.1	+	160.0 ± 48.2	+	0.011	123.2 ± 16.1	+	110.1 ± 5.1	+	NS
Rod-cone a-wave	169.8 ± 45.2	+	191.5 ± 57.3	+	0.009	23.5 ± 3.4	+	23.7 ± 2.4	+	NS
Rod-cone b-wave	391.0 ± 64.1	+	429.8 ± 92.4	+	NS	48.5 ± 2.2	+	47.4 ± 3.0	+	NS
OPs wave index	67.4 ± 15.6	+	83.4 ± 39.0	+	0.012					
Cone a-wave single flash	23.1 ± 4.4	+	25.7 ± 6.3	+	NS	14.4 ± 1.1	+	13.2 ± 0.9	+	NS
Cone b-wave single flash	73.4 ± 21.8	+	96.3 ± 29.5	+	0.008	28.2 ± 2.2	+	29.2 ± 1.5	+	NS
Cone 30-Hz flicker	50.6 ± 10.7	+	69.8 ± 24.1	+	0.009	31.5 ± 4.7	+	28.0 ± 2.3	+	0.005

Normally distributed data: values are mean ± SD

*NS* not significant (*p* > 0.0125), *N* normal distribution

In the ERG, a statistically significant reductions of amplitudes of waves in patients with AF were revealed; scotopic b-wave 24 dB (Ab = 140.9 vs. 160 μV; *p* = 0.011), scotopic a-wave 0 dB (Aa = 169.8 vs. 191.5 μV; *p* = 0009), photopic single flash b-wave (Ab = 73.4 vs. 96.3 μV; *p* = 0.008), photopic flicker response (Ab = 50.6 vs. 69.8 μV; *p* = 0.009), OPs WI = 67.4 vs. 83.0 μV (*p* = 0.012). The increase in peak time of the photopic flicker b-wave (31.5 vs. 28 ms; *p* = 0.005) was observed. Other parameters of the PERG/PVEP/ERG were not statistically significant in comparison with the control group (PERG/PVEP data not shown). Patients with pathological records of the ERG/PERG/PVEP did not have more severe disease (AF) in comparison with ones with normal results.

Three months after the ablation procedure, complete electrophysiological tests (ERG/PERG/PVEP) were performed (*n* = 12 eyes, six men, six women). The mean age was 65.4 years old ± 6.0 (range of age 53–72 years). Four patients (*n* = 8 eyes) had treated arterial hypertension; one (*n* = 2 eyes) was a smoker. No other systemic diseases were observed. Best-corrected visual acuity was 0.79 ± 1.4.

**Fig. 3:**
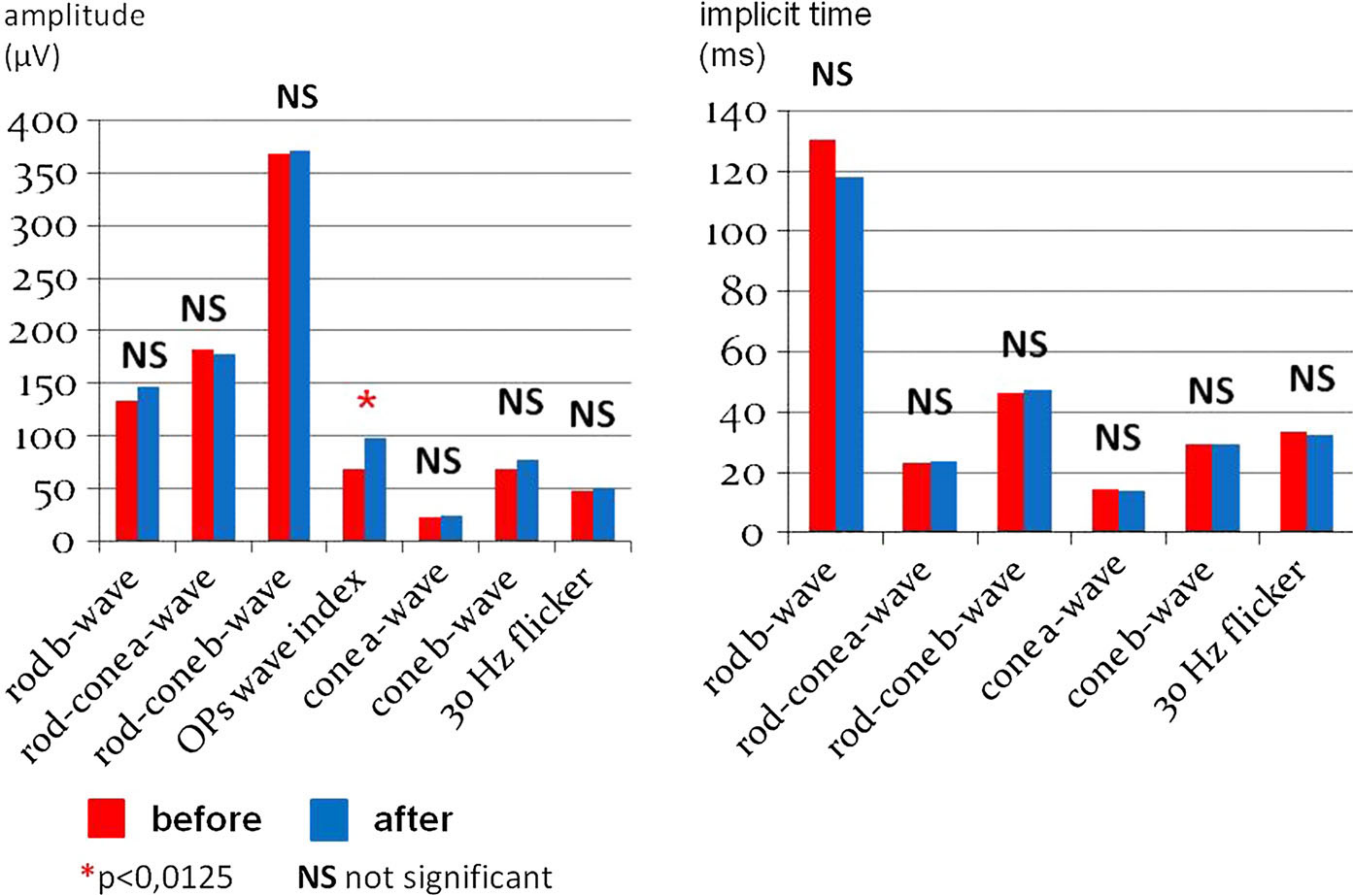
Full-field electroretinogram—eyes of patients with AF before and after ablation (*n* = 12)

The only statistically significant change was an increase in the OPs WI: 97.6 versus 68.3 μV (*p* = 0.002; Fig. 2). Other ERG (Fig. 3; Table 2), PERG and PVEP (data not shown) parameters revealed no statistically significant changes. It has not been shown that patients with initially pathological ERG/PERG/PVEP records were exposed to greater risk of vision loss (*p* > 0.0125). Before ablation, the pathologic OPs WI were found in 24/32 eyes (75 %) (<−2SD for control group). After the treatment, the OPs WI increase to the normal values (−2SD < *x* < 2SD) in 14/24 (58 %) of the previous abnormal recordings were achieved. A greater response was observed in case of younger patients: under 65 years old 10/13 (76 %), in comparison with older ones–4/11 (36 %) after 65 years old.

**Table 2:** Full-field electroretinogram—eyes of patients with AF before and after ablation (*n* = 12)

	Amplitude (µV)				*p* value	Peak time (ms)				*p* value
	AF ablation					AF ablation				
	Before	*N*	After	*N*		Before	*N*	After	*N*	
Rod b-wave	133.3 ± 101.7	+	146.6 ± 115.2	+	NS	130.6 ± 18.1	+	118.1 ± 17.8	+	NS
Rod-cone a-wave	182.5 ± 41.6	+	177.8 ± 55.3	+	NS	23.4 ± 2.2	–	23.9 (95 % CI 21.4–25.1	–	NS
Rod-cone b-wave	368.5 ± 69.1	+	371.4 ± 103.4	+	NS	46.6 ± 2.3	+	47.2 ± 2.4	+	NS
OPs wave index	68.4 ± 15.0	+	97.6 ± 14.4	+	0.002					
Cone a-wave single flash	23.1 ± 3.9	+	23.7 ± 4.8	+	NS	14.2 ± 2.0	+	13.9 ± 1.5	+	NS
Cone b-wave single flash	68.7 (95 % CI 55.6–80.7)	−	77.2 ± 31.1	+	NS	29.3 ± 1.0	+	29.3 (95 % CI 27.7–31.0)	−	NS
Cone b-wave 30-Hz flicker	48.0 ± 12.3	+	50.2 ± 13.1	+	NS	33.6 ± 7.2	+	32.6 (95 % CI 29.2–35.9	−	NS

Normally distributed data: values are mean ± SD. Non-normally distributed data: values are mean (95 % confidence interval, 95 % CI)

*NS* not significant (*p* > 0.0125), *N* normal distribution

## Discussion

The present study has shown the influence of cardiac arrhythmia on the electrophysiological function of the eyes.

In the ERG, a statistically significant reduction in the OPs WI, scotopic a-wave and photopic/scotopic (24 dB) b-wave amplitudes were revealed. The increase in peak time of the photopic flicker b-wave was observed as well. The reduction in the scotopic (0 dB) b-wave and photopic a-wave was present, but was not statistically significant. It is known from literature that a-wave is generated mainly from photoreceptors [24], and b-wave from bipolar cells [25, 26]. The oscillatory potentials (OPs) are small rhythmic waves superimposed on the ascending b-wave of the ERG, and the amacrine and bipolar cells are directly or indirectly involved in their generation [27]. Presented data suggest that dysfunction was present not only in the outer (photoreceptors), but also in the inner retina (bipolar/amacrine cells). This may indicate that the size of retinal dysfunction was greater than assumed. As mentioned in the introduction, the outer layers of the retina are more sensitive to ischemia, than the inner ones. The lowest level of oxygen [28] and pH [29] is reported at the level of the outer nuclear layer under scotopic conditions. It is associated with photoreceptors’ (mostly rods’) high energy/oxygen demands and specific circulation determinants. In the hypoxia, photoreceptors receive blood from the choroid-90 % and the retinal vessels-10 % (both dark and light adaptation) [12]. Although the choroid has one of the highest blood flows in the body, it has no effective autoregulation [16, 17]. In the contrast, retinal circulation (central retinal artery) is highly dependent of the blood concentration of O_2_, CO_2_ and pH levels in the inner retina [6, 16, 28]. A retinal flux can be increased in hypoxia by up to 336 % [6], which is not possible in the choroid. Moreover, as a response to the outer retina higher energy/oxygen consumption in the scotopic conditions, retinal flow may be increased by 40–70 % [29] and 67 % [30]. In comparison with the photopic conditions, in scotopic conditions photoreceptors oxygen consumption increases from 1.4 to 5.1 ml O_2_/100 g min [11–13]. The inner layers of the retina consume oxygen at a constant level, regardless of the light conditions: 1.47–4.6 ml O_2_/100 g min [13, 30–32]. This level is comparable to the oxygen consumption in the brain (2–5 ml O_2_/100 g min) [8]. The retinal dysfunction in AF patients occurred probably due to ischemia. Retinal ischemia usually affects the ERG peak latencies as well as the amplitudes. However, the rod-cone a-wave and cone b-wave amplitude reduction is not accompanied by an increase in peak time. This is probably due a large standard deviation of the patients’ responses with respect to controls (respectively, before ablation: cone a-wave SD 3.4 vs. 2.4 ms; cone b-wave SD 2.2 vs. 1.5 ms). Larger standard deviations were observed in the group of patients <65 years old. In the group >65 years old, the responses were more consistent and the amplitude reduction was accompanied by an increase in peak time. This may indicate a larger occurrence of ischemic changes in this group of patients. In the case of animal studies, it was found that the most sensitive indicator to ischemia was as follows: amplitude of b-wave [19, 33–35], amplitude of the scotopic oscillatory potentials, photopic flicker response [36–38], c-wave [19]. In human studies, scotopic OP amplitude [39, 40] and peak time [41] have been reported to be most affected by retinal ischemia. An ischemic etiology of electrophysiological abnormalities observed in patients with AF is supported by the fact that similar changes occur in other vascular diseases like hypertension [42, 43], diabetes mellitus [27] and carotid stenosis [27].

The function of retinal ganglion cells (RGC) in patients with AF was preserved. In the PERG test, no statistically significant changes in amplitudes and peak times of waves P50/N95 were found. These data are consistent with reports that the PERG factors are less susceptible to ischemia in comparison with the full-field ERG parameters [36]. In the PVEP tests in case of AF patients, no statistically significant results were obtained. However, a trend of an increase in P100-wave peak time after small check stimulation was observed (0°16′). There were no similar changes after the stimulation of large check (1°4′). The increased P100 peak time is not characteristic for optic nerve ischemia, contrary to reduced P100 amplitude [44, 45]. The optic nerve and RGC are both supplied with central retinal artery. It is suggested that high flux and good autoregulation mechanisms cause that these structures to be less susceptible to ischemia than the outer segments of the retina.

Influence of the cardiac arrhythmia treatment on the electrophysiological function of eyes has never been assessed. However, there have been numerous studies evaluating the positive effect of carotid stenosis treatment on the eye function [46]. An increased blood flow in the ocular vessels assessed in Doppler ultrasonography after such intervention is reported [46, 47]. It is possible that after AF ablation, similar mechanism at the level of retinal circulation may occur. After treatment, the trend of an increase in the amplitudes of P50 and N95 in the PERG test, the shortening of latency of P100 in the PVEP was observed. These results were not statistically significant, which may be due to the limited sample size (*n* = 12). In the full-field ERG, only the OPs WI has significantly improved after AF ablation (*p* = 0.002) (Fig. 3; Table 2). According to some authors, OPs are a good tool to detect early signs of circulatory disorders [48, 49]. However, a clear trend of scotopic/photopic b-wave amplitude increase of 10 and 12 %, respectively, was also observed. This may reflect the improvement of bipolar cell function. In the outer retina, the improvement was less evident (a-wave parameters). Better function of amacrine (validated) and bipolar cells (suggested) may confirm that the AF ablation may favorably affect the inner retina. Probably it is associated with a better blood supply and self-regulation of flows in the inner layers in comparison with the outer ones. That may explain, why effects of cardiac arrhythmia normalization can be seen more clearly in amacrine cells function, contrary to photoreceptors’ one, which was damaged both of them before and after ablation. It is worth noting that after the ablation procedure, only the OPs values were normalized (OPs VI = 97.6 µV after treatment vs. OPs VI = 83.4 µV controls). Other parameters, the a-wave and b-wave, despite the described improvement did not reach the level of the control group (“AF after treatment” Table 2 vs. “Controls” Table 1). In the authors’ opinion, this may indicate the existence of a persistent retinal hypoperfusion resulting from atherosclerotic changes. A cardiac cause of the hypoperfusion was excluded based on the normalization of heart rate and normal ejection fraction by ultrasound.

Before an ablation, the pathologic OPs WI was found in 75 % of eyes. After the treatment, normalization was observed in 76 % of eyes in patients under 65 years old, while only 36 % in case of above 65 years old. Presented OPs WI differences are probably caused by less advanced atherosclerosis among younger patients. It is suggested that this group is more likely to improve after the treatment. It seems reasonable that patients with persistent pathological OPs after ablation are at greater risk of vascular complications in the future, because of more advanced atherosclerosis. Therefore, this group of patients should undergo more careful and regular follow-ups, both cardiological and ophthalmological.

## Conclusions

1. In an analyzed series of AF patients’ retinal dysfunction was presented and detected in the ERG test.
2. The AF ablation may improve a retinal function, which was registered on OPs WI.
3. The OPs WI has a potential value in the estimation of the effectiveness of AF ablation.
